# A spiking neural network model of self-organized pattern recognition in the early mammalian olfactory system

**DOI:** 10.3389/fncir.2014.00005

**Published:** 2014-02-07

**Authors:** Bernhard A. Kaplan, Anders Lansner

**Affiliations:** ^1^Department of Computational Biology, School of Computer Science and Communication, Royal Institute of TechnologyStockholm, Sweden; ^2^Stockholm Brain Institute, Karolinska InstituteStockholm, Sweden; ^3^Department of Numerical Analysis and Computer Science, Stockholm UniversityStockholm, Sweden

**Keywords:** pattern recognition, olfactory bulb, piriform cortex, large-scale neuromorphic systems, spiking neural network, BCPNN, concentration invariance, pattern rivalry

## Abstract

Olfactory sensory information passes through several processing stages before an odor percept emerges. The question how the olfactory system learns to create odor representations linking those different levels and how it learns to connect and discriminate between them is largely unresolved. We present a large-scale network model with single and multi-compartmental Hodgkin–Huxley type model neurons representing olfactory receptor neurons (ORNs) in the epithelium, periglomerular cells, mitral/tufted cells and granule cells in the olfactory bulb (OB), and three types of cortical cells in the piriform cortex (PC). Odor patterns are calculated based on affinities between ORNs and odor stimuli derived from physico-chemical descriptors of behaviorally relevant real-world odorants. The properties of ORNs were tuned to show saturated response curves with increasing concentration as seen in experiments. On the level of the OB we explored the possibility of using a fuzzy concentration interval code, which was implemented through dendro-dendritic inhibition leading to winner-take-all like dynamics between mitral/tufted cells belonging to the same glomerulus. The connectivity from mitral/tufted cells to PC neurons was self-organized from a mutual information measure and by using a competitive Hebbian–Bayesian learning algorithm based on the response patterns of mitral/tufted cells to different odors yielding a distributed feed-forward projection to the PC. The PC was implemented as a modular attractor network with a recurrent connectivity that was likewise organized through Hebbian–Bayesian learning. We demonstrate the functionality of the model in a one-sniff-learning and recognition task on a set of 50 odorants. Furthermore, we study its robustness against noise on the receptor level and its ability to perform concentration invariant odor recognition. Moreover, we investigate the pattern completion capabilities of the system and rivalry dynamics for odor mixtures.

## 1. Introduction

The major task of the olfactory system is to perform recognition of odors which is essential for survival by identifying edibility or danger. An odor evokes spatio-temporal patterns of activity in different stages of the olfactory hierarchy. The crucial mechanisms involved in odor object recognition are widely unknown, which is mainly due to the complexity of interactions and the transformations of information occurring between the different stages. In order to study the mechanisms embedded in the olfactory system, a system-level approach is required, comprising the three major levels of the early olfactory hierarchy including the epithelium, where the stimulus enters the nervous system, the olfactory bulb (OB) where the first transformation happens, and the piriform cortex (PC) which integrates and stores the information relevant for odor recognition (Gottfried, 2010; Wilson and Sullivan, 2011), and decision making (Gire et al., 2013). The OB and the PC and the connectivity between the two are crucial components for solving pattern recognition tasks, however experiments are only beginning to shed light on the possible connectivity principles. Neurons in the PC receive convergent synaptic input from different glomeruli (Apicella et al., 2010), but the question as to which principles underlie the connectivity between OB and PC is not yet resolved.

In this study we try to bridge the gap between the biophysics seen from a detailed perspective and the organization principles on a system level. Here, we present a model which is able to recognize artificial odor patterns in a self-organized manner using a Hebbian–Bayesian learning rule and ideas inspired from machine learning implemented on a biophysically detailed substrate. We will first embed our study in the context of existing literature, before we will explain the goals and hypotheses of our study.

### 1.1. Context and overview of existing primary literature

The olfactory system has long been a model system to study memory formation (Haberly and Bower, 1989; Brennan et al., 1990), object recognition (Davis and Eichenbaum, 1991) and pattern completion (Barnes et al., 2008). Computational modeling of the olfactory system began with the work by Rall et al. (1966) and continued to complement experimental research by testing hypotheses under controlled conditions and by connecting behavior with the underlying mechanisms.

Many studies focus on a single component of the lower levels of olfactory processing hierarchy, e.g. the OR responses (Hopfield, 1999), the epithelium (Simões-de Souza and Roque, 2004b; Sandström et al., 2009a), the OB or subparts thereof (Anton et al., 1991; Davison et al., 2003; Sandström et al., 2007; Brea et al., 2009; Linster and Cleland, 2009; Li and Cleland, 2013; Yu et al., 2013). There have only been few studies that attempt to model multiple parts of the olfactory pathways, for example, the study by Simões-de Souza and Roque (2004a) combines epithelium and OB. Modeling work on the PC can have a high level of detail (Wilson and Bower, 1992; Vanier, 2001) and describes the PC as a content-addressable memory system that is optimized for storing synaptic representations of odors through Hebbian learning (Barkai et al., 1994), yet often lacks a fair representation of the lower parts of the sensory pathway and the interactions in between. On the intermediate scale, Freeman's K-sets (Freeman and Erwin, 2008) have been used to model pattern recognition with chaotic dynamics (Yao and Freeman, 1990; Li et al., 2005), but this approach does not explain how connectivity emerges and misses lower parts as well. More recently, computational studies connect function with self-organization mechanisms and emergent connectivity in the OB (Migliore et al., 2007; Linster and Cleland, 2010; Migliore et al., 2010). The model by Li and Hertz (2000) involves both OB and PC and is based on rather abstract, oscillatory units and recognition works on the basis of temporal characteristics, which is argued for by other studies as well (Hopfield, 1991, 1995; Margrie and Schaefer, 2003; Schaefer and Margrie, 2012; Brody and Hopfield, 2003). Whether the temporal coding is crucial for recognition is up for debate and we will come back to this question in the discussion. Linster et al. (2009) presents a small scale model comprising simple models of olfactory receptor neurons (ORNs), MT, PG, granule and PYR cells to study response habituation effects based on synaptic adaptation and potentiation in PC for single odor patterns. The study offers a comparison with behavioral data, but lacks the generic pattern recognition capabilities which we are addressing in this study.

There exist a number of studies on classification and recognition in the insect olfactory system (Huerta et al., 2004; Nowotny et al., 2005; Schmuker and Schneider, 2007; Schmuker et al., 2011). The study by Nowotny et al. (2005) uses an approach similar to ours, by transforming the combinatorial code in the antennal lobe (the equivalent of the OB in insects) into a higher dimensional space and applying Hebbian learning with mutual inhibition in the mushroom body (the PC equivalent in insects). An improved understanding of the olfactory system through modeling also lead to substantial advances in machine olfaction (Gutierrez-Osuna, 2002; Pearce et al., 2006; Raman et al., 2011).

### 1.2. Purpose of this study

As we have outlined above, most existing models either use an abstract description with components far away from the biological substrate or have a high level of detail but lack other relevant system components leading to an incomplete picture of the olfactory system. Furthermore, the role of the different components from a computational perspective is still under debate, for example whether most of the transformations involved in pattern recognition take place in the OB or rather in the PC and how the interactions between the two is organized is unknown. What is lacking is a generic computational model capable of behavioral relevant functions like pattern recognition which involves the ability to self-organize and which is able to run in a biophysically plausible setting. In this work, we are trying to make a first step toward filling this gap by presenting—to the best of our knowledge—the first functional biophysical model of the olfactory system integrating the first three stages on a high level of detail.

The goal of this paper is threefold. First, we propose a generic approach for neural information processing that generates the connectivity from the OB to the PC and within the PC by means of self-organization and competitive learning. More generally, we model the activity dependent formation of connectivity between sensory layers and cortical memory systems as well as the recurrent long-range intra-cortical connectivity. Second, we show that a biophysically plausible implementation of this approach in the context of olfaction is feasible. Third, we prove the functionality of our concept and the spiking implementation in a number of pattern recognition tasks and study the system's behavior therein.

Our model is based on an abstract generic model for cortical information processing (Lansner et al., 2009; Persaud et al., 2013) which offers a recursively applicable algorithm to generate functional connectivity within and between processing stages and is realized as a multi-layer spiking neural network. Furthermore, we explore the possibility of an OB model making use of a concentration interval code in the mitral (MT) cell layer to serve as input to an attractor network model of the PC, and we investigate the behavior of the system in the five following tasks. First, we show the functionality in a pattern recognition task for 50 artificial odor patterns. Second, we test the ability of the system to recognize odors at different concentrations and propose a solution to the concentration invariance problem (Cleland et al., 2011) in olfaction. In the third task we challenge the system with noisy patterns mimicking impure odors. The fourth task shows the system's pattern completion capabilities by testing with incomplete patterns of different sparsity, and the fifth task is to distinguish between different mixtures of learned patterns.

### 1.3. Main hypotheses

We will now explain the main computational hypotheses on which the model is based, name important experimental findings supporting these and explain the implementation in section 2. Hereby we move the olfactory pathway along from the receptor level to the cortex.

#### 1.3.1. Activity dependent connectivity from epithelium to bulb

Each ORN expresses only one olfactory receptor (OR) (Buck and Axel, 1991), and each odorant activates a broad range of ORNs involving different ORs (Firestein, 2001). ORNs expressing the same OR (in the following named an ORN-family) have different sensitivities to the same odorant and show dose-response curves with activation thresholds and saturation points covering a broad dynamic range (Grosmaitre et al., 2006). An ORN-family projects to only one or two glomeruli (Vassar et al., 1994; Mombaerts et al., 1996). We extend these principles by adding our first hypothesis which affects the connectivity from ORNs to OB. We assume that axons from one ORN-family undergo an activity-dependent sorting process when connecting to the dendritic trees of MT and PG neurons in the same glomerulus. This assumption extends the chemoaffinity hypothesis (Sperry, 1963) and applies the existing idea that activity and experience is involved in the axon growth process (Gill and Pearce, 2003; Tozaki et al., 2004; Kerr and Belluscio, 2006; Imai and Sakano, 2008; Sakano, 2010; Mori and Sakano, 2011) to the local axon sorting process (Zhao and Reed, 2001; Serizawa et al., 2006; Takeuchi et al., 2010) and thereby shapes the response properties of MT cells. This activity-dependent sorting activates MT belonging to the same glomerulus as a function of the average firing rate of the convergent ORNs, an idea picked up earlier by Anton et al. (1991); Cleland and Linster (2005). A previous study has shown that activity dependent sorting can lead to map formation in the OB which could have perceptual advantages (Auffarth et al., 2011). We are using axon sorting mechanisms that are possibly active within an ORN-family to implement our second hypothesis, a concentration interval code in the OB.

#### 1.3.2. Concentration coding

The concentration interval coding hypothesis assumes that each MT cell has one preferred concentration of an odor to which it responds maximally (Sandström et al., 2009b) and we will explain in detail in section 2 how these two hypotheses are used to implement a fuzzy concentration interval code in the OB. This hypothesis is inspired by the idea of neuronal tuning which assumes that neuronal responses are tuned to specific inputs through experience and rules for optimally covering the stimulus space have been studied (Zhang and Sejnowski, 1999; Brown and Bäcker, 2006). Cells coding for an interval of a certain stimulus dimension have been found in many sensory systems. For example, just to name a few examples, in vision there exist interval codes for orientation (Hubel and Wiesel, 1962; Schoups et al., 2001; Li et al., 2012) and direction (Albright, 1984), in the auditory system for pitch (Bendor and Wang, 2005), and position, direction, speed (Poirier et al., 1997), in hippocampus place or grid cells show strong responses to their preferred position (Moser et al., 2008), and in the motor system neurons are tuned to end positions of movements and other parameters (Aflalo and Graziano, 2006).

The interval coding strategy can be used to encode variables in a probabilistic way, as tuning curves of individual neurons overlap and the value encoded by a population of units can be decoded in a Bayesian optimal sense (Ma et al., 2006). This “fuzzy” coding is related to the concept of Gaussian Mixture Models (GMMs), a generic probabilistic model capable of representing arbitrary densities which makes this coding suitable for unsupervised classification algorithms. GMMs are well-established for coding in learning and classification systems for complex stimuli, e.g. speaker recognition (Reynolds et al., 2000), person identification (Stylianou et al., 2005), and image classification (Permuter et al., 2003).

One of the canonical computations believed to be performed by lower sensory areas is decorrelation (Cleland, 2010; Linster and Cleland, 2010), which we assume to be performed in the concentration domain by MT cells receiving input from the same glomerulus (so called sister MT cells). We thereby assume that cells connected to one glomerulus operate as functional modules making use of the columnar organization as revealed by a viral tracer study (Willhite et al., 2006). In this study, we apply this idea to encode odorant concentration in a fuzzy manner by MT cells and explore the possibility of such a code in a functional model for self-organized pattern learning. The advantage of this coding scheme is that odor identity and concentration can be represented at the same time without relying on precise spike timings.

Whether mitral cells do exhibit a concentration interval code or not is not fully resolved, due to contrary indications from different experiments and the complex temporal dynamics of alternating excitation and inhibition (Chaput et al., 1992) and their sensitivity to concentration (Chalansonnet and Chaput, 1998). Experiments by Tan et al. (2010) show that at least in some glomeruli mitral cells do not exhibit a concentration interval code as we propose here. Other studies, in contrast, report non-monotonous firing rates for increasing concentrations in mice (Reinken and Schmidt, 1986), rats (Wellis et al., 1989), and hamsters (Meredith, 1986). The study by Egana et al. (2005) suggests that sister MT cells often exhibit very different response characteristics in terms of increase in firing rate due to odor exposure and their respiratory-related temporal patterns. Likewise, it has been shown that sister MT cells show non-redundant temporal behavior (Dhawale et al., 2010) and it has been suggested that the reason for that might be found on the circuit level. Bozza et al. (2004) used an imaging technique showing the synaptic vesicle fusion in ORNs targeting glomeruli and found different concentration-response relationships for different glomeruli. The most sensitive glomeruli to 2-hexanone showed saturated response curves at an intermediate concentration (see Figure 5E in Bozza et al., 2004), thus providing non-monotonous input into some glomeruli which could possibly explain the different experimental indications mentioned above. The response characteristics of bulbar neurons have been studied mostly in anesthetized animals, but recent experiments by Kato et al. (2012) show that mitral and granule cell react differently toward anesthesia, and odor representations are different in awake and anesthetized states. Hence, MT cell odor responses might be more narrowly tuned in unanesthetized animals and strongly depend on the behavioral context (Shipley et al., 2008). Here, we explore the possibility of this hypothetical coding scheme in a biophysically detailed model and explore the capability for concentration coding in a functional context from a systems level perspective. An alternative idea, which is not mutually exclusive to the concentration interval coding hypothesis, is that MT cells code odor concentration and odor identity by the spike latency within a sniff (Margrie and Schaefer, 2003; Schaefer and Margrie, 2012). We will discuss the spike latency coding hypothesis in section 4 in the context of our results.

#### 1.3.3. Rate-based hebbian learning from OB to PC

Our next hypotheses concern the mechanisms underlying projections from OB to PC. First, we assume that learning is rate-based and hence primarily taking place on a coarser time-scale than e.g. spike-timing dependent plasticity usually modeled on a timescale of milliseconds, but use the response of the OB to odorant patterns over one long sniff (modeled as one long inhalation leading to a stimulus of ~400 ms and simulated for 1600 ms). Furthermore, we do not regard learning mechanisms active within the OB, e.g. MT responses changing with exposure (Fletcher and Wilson, 2003), generation of granule cells (Mandairon et al., 2006) and disregard the dynamics of the odor afterimage (Patterson et al., 2013). We assume that the main component in olfactory learning is how projections from OB to PC and within PC are created and that pattern recognition is based on the activity evoked through these afferent fibers terminating in the PC and the recurrent activity within the PC. In order to organize the connectivity from OB to PC we use the mutual information of normalized individual mitral cells responses and a competitive correlation-based learning mechanism, which is used as input to the Bayesian Confidence Propagation Neural Network (BCPNN) algorithm (Lansner and Ekeberg, 1989; Lansner et al., 2009). Similar implementations thereof have been applied in various setups (Sandberg et al., 2002; Lansner et al., 2003, 2009; Auffarth et al., 2011; Persaud et al., 2013).

Oscillations are a prominent phenomenon in the olfactory system. In this study, we do not study oscillations, as they do not play a crucial role within our framework for the pattern recognition tasks we consider and, because according to our hypothesis, learning takes place on larger time-scales than oscillations do occur. Hence, oscillatory signatures have not been analyzed in this study, but can be found in modular network of very similar type as ours as studied by Lundqvist et al. (2010, 2011).

#### 1.3.4. Olfactory cortex as an attractor memory system

Another important component of the olfactory memory system is the recurrent connectivity within the PC. The association fiber network prominent in PC is regarded as the substrate for a content addressable and distributed memory system (see Haberly, 2001; Wilson et al., 2006; Wilson and Sullivan, 2011 for reviews). Our cortex model is inspired by the idea that the olfactory cortex acts like other associative cortices in the sense that it learns to create and distinguish sparse and distributed representations of odor patterns, and is able to associate simpler odor patterns with each other to form abstract complex odor objects (Haberly, 2001; Wilson and Sullivan, 2011). Attractor networks have been proven to be an effective model to explain memory formation and retrieval (Amit, 1992; Hasselmo and McClelland, 1999) and other brain functions (see e.g. Rolls, 2008) and are one approach to implement higher cognitive functions like holistic perception in biophysically detailed simulations (Lansner, 2009). Inspired by previous models, we see the cortex as a crucial part in the pattern classification process and derive the projections from OB to OC and the recurrent cortical connectivity with the help of the BCPNN algorithm (Fransén and Lansner, 1998; Sandberg et al., 2002; Lansner et al., 2009).

### 1.4. Principle approach

This study explores the possibility to apply a generic, recursive approach to a self-organized pattern recognition system on a biophysical substrate resembling the mammalian olfactory system. Despite the fact that the PC is a three-layered paleocortex, we assume the PC to work in a similar way as other sensory and association cortices with regard to memory formation. In the model design and choice of parameters, we put emphasis on functional implications and on a qualitative match to the biological substrate rather than an accurate quantitative agreement between simulations and experimental data. Thus, our approach should not be seen as realistic in all detail, but rather be regarded as explorative and plausible toward bridging the gap between system-level computations and biophysical detail. We use numerical simulations of single and multi-compartment neuron models described by the Hodgkin–Huxley formalism and apply rate-based learning rules to derive functional connectivity to support pattern recognition. We used this family of neuron models, for several reasons. First, there already exists a number of neuron model implementations for the most prominent bulbar and cortical cell types that are relevant for our approach and ready to use with the NEURON simulator (Hines and Carnevale, 1997). Second, neuron models that were not implemented at the beginning of the studies could be adapted from existing neuron models (see Table 1 for a brief overview of neuron types). Third, network models in NEURON are easily parallelizable and hence can be extended to larger scales and offer the possibility for future refinements and extensions, e.g. if more biophysical realism is desired.

**Table 1:** **Neuron and synapse models and choice of parameters**.

**Neuron name**	**Type**	**Stage**	**Number of compartments**	**References**
ORN	Exc	Epithelium	1	Adapted from Garcia (2010)
MT	Exc	OB	4	Davison et al., 2003
PG	Inh	OB	3	Davison et al., 2003
Granule cell	Inh	OB	3	Davison et al., 2003
PYR	Exc	PC	1	Adapted from Pospischil et al. (2008)
RSNP regular spiking	Inh	PC	1	Adapted from Pospischil et al. (2008)
Basket cell (fast spiking interneuron)	Inh	PC	1	Adapted from Pospischil et al. (2008)
Readout neuron		PC	1	Adapted from Pospischil et al. (2008)

## 2. Materials and methods

### 2.1. Neuron and synapse models and choice of parameters

In order to model a multi-layered network with a reasonable level of detail, one has to fill several gaps by making assumptions because many aspects and parameters of the real system are not known. We have tried to use realistic parameters wherever possible, but as the primary goal of this paper is to present a holistic architecture implementing a high-level task with a spiking neural network, we had to reduce this goal at several points to achieve the desired function.

#### 2.1.1. Neuron types

For all simulations we use neuron models described by the Hodgkin–Huxley formalism, an overview of the used neuron models is shown in Table 1. Our principle approach was to use existent neuron models without modification if possible and to adapt existing neuron models if the desired function required changes. For ORNs we have extended an existing single-compartmental neuron model described in Pospischil et al. (2008) by adding a time-dependent input current to model the odor stimulus, a low- and a high-threshold Calcium current and a Calcium activated Potassium channel to provide adaptation mechanisms to guarantee saturating dose-response curves. The ORN channel conductances have been tuned so that the model shows plausible dose response curves for a family or ORNs, i.e. different response onsets depending on the sensitivity and saturating output rates for high stimulus concentrations.

In the OB, we use three multi-compartmental cell types: MT cells, granule cells and PG neurons. As in other studies we model mitral and external tufted cells as one neuron type, as our focus lies in the projection from both neuron types to the cortex. Neuron models for MT and granule cells are identical to those in the study by Davison et al. (2003). MT cells have compartments for glomerular dendrite, primary dendrite, soma and secondary dendrite connecting to granule cells. Granule cells have compartments for their soma, peripheral and deep dendrites. In the absence of a neuron model for PG cells at the beginning of our study, we used the same neuron model for PG as for granule cells using their peripheral dendrite for interactions with ORNs and MT cells and dendrodendritic interactions to convey PG output to MT cells.

The PC model contains one excitatory adapting neuron type (PYR), a fast-spiking inhibitory interneuron [in the following called basket cell (Ekstrand et al., 2001)] and a regular spiking non-pyramidal (RSNP) neuron (all adapted from Pospischil et al. (2008). The BCPNN algorithm as described later gives bias values for each cortical module, which can be interpreted as intrinsic excitability implemented as an inhibitory A-type Potassium current (Bergel, 2010) added to RSNP and PYR neurons.

#### 2.1.2. Synapse models

Excitatory synapses are realized through exponential currents mediated by AMPA receptors with a time constant of 10 ms and NMDA receptors implemented as in Davison et al. (2003), which models a Magnesium block and operates at a longer time constant (≈150 ms). Inhibitory synapses only have one time scale and are modeled as exponential currents mediated by GABA receptors with a time constant of 20 ms.

#### 2.1.3. Choice of parameters

One set of parameters determines the network size that needs to be adapted to the number of patterns the system is trained with. These are the number of glomeruli (equal to the number of ORs), the number of HCs and the number of MCs per HC. We have not explored the number of ORs, HCs and MCs required to successfully learn a given number of patterns, because this would be out of the scope of this paper and should be studied with a less detailed model.

The BCPNN algorithm yields the connectivity between the OB and OC and within the OC as “abstract weights”. Hence, these parameters are estimated by BCPNN, whereas the translation into biophysical weights is done with the help of free scaling parameters that were chosen to yield biophysically plausible synaptic conductance values in the order of a few nS. Furthermore, there exists a large set of model parameters (on the order of 70) controlling various aspects, like the individual cell models (cell morphology, ion channel conductances, background noise), connectivity parameters from ORNs to OB and within the OB. A subpart of these have been tuned by hand to achieve the desired behavior.

Because of the complexity of this model and the immense number of parameters involved, we omit a list of parameters here, but refer to the existing literature and the simulation code, which is available on request. As already mentioned, the focus of this study is to implement a functional model operating on multiple stages and not to build a precisely matched counterpart of the biological substrate. Hence, we decided to choose parameters to fulfill functional requirements as this is our primary goal. In combination with the small size of the networks compared to real systems this might have lead to unrealistic values in some cases. Furthermore, the vast amount of parameters would make a parameter sensitivity analysis extremely complex and computationally intensive and as the parameter space is very high-dimensional, it is likely that many different operating regimes could be found.

Simulations were performed with the NEURON simulator (Hines and Carnevale, 1997) on a Cray XE6 system using 96–120 cores. For setting up simulation preparation, connectivity and analysis of results we used python with the modules numpy (Oliphant, 2007), scipy (Jones et al., 2001–2013) and orange (Demšar et al., 2013). Figures and data visualization were done using matplotlib (Hunter, 2007) and Inkscape (Andler et al., 2004–2014). Cell parameters were identical for all neurons of the same type. To account for natural variability all weights were randomly modified by 10%, the initial membrane voltage was drawn from a normal distribution with mean −70 mV, and standard deviation 5 mV, and each neuron (except readout neurons) received Poisson spike trains as background noise to model both network effects and stochastic opening and closing of ion channels.

### 2.2. Odor input patterns

In order to decide how strong each family of ORNs (each expressing one OR and targeting only one glomerulus) gets activated by an artificial odor pattern, we derive a distribution of odorant-OR affinities based on real-world data. Haddad et al. (2008) presented an optimized set of 32 physico-chemical descriptors which could account for variability in neural responses of ORNs and glomeruli in different species for different sets of odorants. This gives a 32-dimensional space, in which the 447 odorants they provide can be described. In short, we place virtual ORs in this 32-dimensional space as centroids resulting from clustering the odorants, calculate the Euclidean distance between the virtual ORs and real-world odorants, and based on this distance we obtain the affinity between the OR-odorant pair. This approach is inspired by the odotope theory Shepherd (1987); Mori (1995), which suggest that the molecular shape of an odorant and the molecular preference of an OR determine the OR response. This idea implicates that spatial proximity of ORs in this multidimensional space implies similar molecular receptive ranges of the ORs. This idea is currently debated because not only functional groups of odor molecules, but also the vibrational energy spectrum of molecules does play a role in determining OR responses (Franco et al., 2011; Gabler et al., 2013). Nevertheless, for simplicity we chose the odotope theory as a guiding principle to generate artificial odor patterns. It should be emphasized that the pattern recognition capability of our system is not constrained to this way of generating artificial odor patterns. Despite the fact that our virtual ORs lack a direct biological correspondence, the presented approach of interpreting ORs as centroids after clustering the odor space seems plausible, assuming that ORs could have specialized to code for parts of the olfactory world. The study by Geisler and Diehl (2002) suggests that perceptual systems are designed for encoding natural stimuli in an optimal way. Nei et al. (2008) suggest that variations in chemosensory receptor gene repertoires among species can be explained to a large extent by the adaptation of organisms to different environments. In the following, we describe the details of our approach inspired by these ideas.

The ORs were chosen to be the centroids of clusters in the odor space computed by the k-means clustering algorithm (Hartigan and Wong, 1979). As the distances between ORs and odorants are based on the results of the clustering procedure and hence depend strongly on the number of ORs to be put in the odor space and the random initial conditions, we have pooled distance distributions for different numbers of ORs over 100 trials. The motivation behind this approach is to get a picture of the real-world odor space and to derive a generic way to generate arbitrary numbers of virtual odor patterns that share the same characteristics in terms of odorant-OR distances as real odors could have based on the odotope idea described above.

For each number of ORs (centroids) we fitted a trimodal normal distribution to the obtained distance distributions, as it resembled the distribution reasonably well (see Figure 2) and observed that the fit parameters did not change qualitatively for distributions when 20–66 centroids were used to cluster the odor space. For more than 66 centroids, the k-means algorithm could often not converge because of too many centroids populating the odorant space and leaving centroids without odorants in their proximity. Hence, we used the averaged fit parameters of the distance distribution for 20–66 centroids to obtain a method to draw distances between artificial odorants and ORs, which gives us an average distance distribution 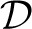 between real world odorants and virtual ORs. The activation pattern of an odorant was generated by first randomly choosing *n*_activated_ ORs that do show a response given the system is exposed to that odorant in a noise-free environment (how noisy patterns are generated will be explained below). For each pattern we chose a random integer *n*_activated_ to be between 30% and 50% of all receptors, as this is in the range of what has been reported experimentally (Ma et al., 2012). For each activated odorant *i* and OR *j* a distance *d*_*i, j*_ was sampled from 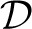 and transformed into an affinity 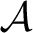
_*i, j*_ by applying this transformation function:

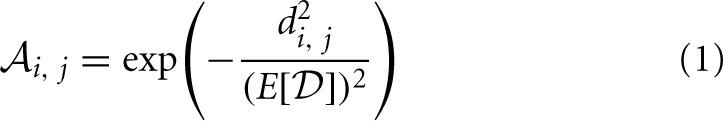



where *E*[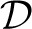] = 7.7 is the expected value for distances sampled from the distribution 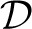 as shown in Figures 2A,B. We chose this transformation function in order to have a strong influence of the distance between odorant and OR in the space determined by Haddad et al. (2008) and to obtain a population of affinity values covering the whole range between 0 and 1 even for small sample sizes of odorant-receptor pairs as in our model simulations. An example set of 50 patterns for 40 ORs is shown in Figure 4A.

The perception in noisy environments was modeled by modifying each element in the affinity matrix 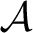 resembling an odorant-receptor pair to 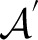:

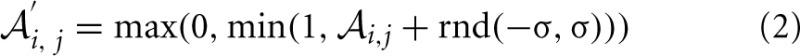



where rnd(−σ, σ) stands for a random number uniformly distributed between −σ and σ, σ stands for the strength of noise. By this means affinities are constrained to the interval between 0 and 1. The idea behind this approach is that in noisy environments, other odors unrelated to the original odor pattern might be present which is represented by having new non-zero elements in 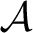, whereas existing OR responses might be suppressed at the same time. For simplicity we have not considered the partly competitive and non-linear interactions between odorants and receptors (Rospars et al., 2008) when a receptor could react to several present odorants.

### 2.3. The olfactory epithelium

The epithelium has been modeled as a population of ORNs without taking the spatial dimension into account. For simplicity, ORNs have been modeled as single-compartment Hodgkin–Huxley neurons with the goal to have a variety of saturating dose-response curves, similarly to experimental studies (see e.g. Rospars et al., 2000, 2003, 2008). An odorant stimulus is modeled as an input current as shown in Figure 2, either as a single puff stimulating ORNs for ~450 ms or as a sequence of four briefer sniffs with a frequency of ~4 Hz. The maximum input current into one ORN is determined by the product between the affinity of the OR expressed by the ORN family to the respective odor and by the maximum excitatory conductance determined by the physiology of cell, which could be the cell size, number of expressed ion channels or the number of receptors on the cilium of the cell. This product of affinity between an OR and an odor, which influences the individual ORN response, can be seen as the fraction of activated receptors or opened ion channels exciting the ORN. This fraction of activated receptors (OAV for odor activity value) can be translated into a concentration *c* or dose (without considering physical units) by applying *c* = OAV / (1 − OAV). Consequently, affinity values (OAV) values are constrained to be between 0 and 1.

We assume here that ORNs expressing the same OR do not have a single value for the maximum conductance, but rather a distribution based on the profound differences in response kinetics as seen in the experimental studies (Rospars et al., 2003; Grosmaitre et al., 2006) and described by statistical population models (Sandström et al., 2009a; Grémiaux et al., 2012). Figure 2C shows the responses of two example receptor neurons to excitatory stimuli. In the simulations presented throughout the study, our model contains 40 populations, each expressing a different OR and comprising 800 neurons that project onto one glomerulus but could be scaled up to include more ORs or more ORNs.

### 2.4. The olfactory bulb

**Figure 1:**
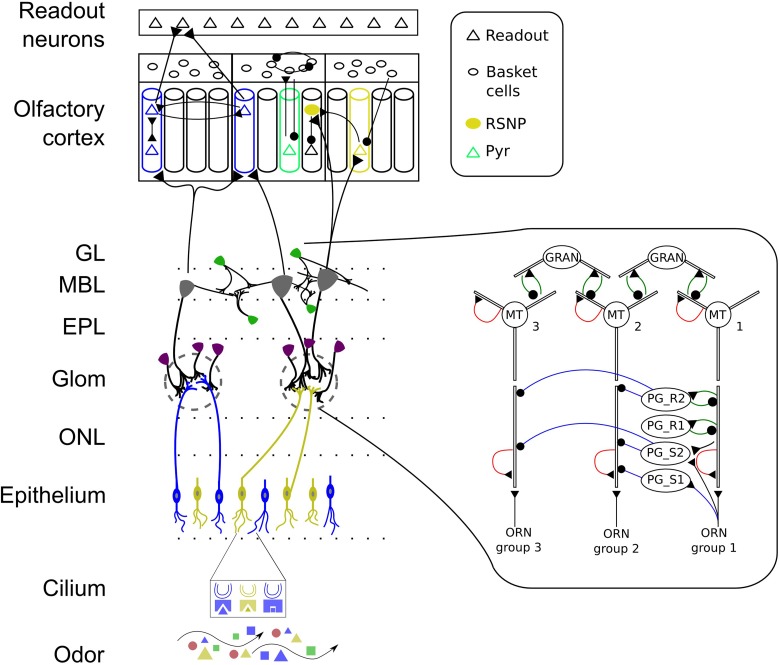
**Schematic of the early stages of the mammalian olfactory system**. Odors bind to receptors in the cilia of ORNs and lead to input currents based on the affinity between odorant and OR and on the ORN sensitivity. ORNs expressing the same OR make excitatory connections with PG and MT cells in one glomeruli. PG cells show different dendritic arborization patterns and interact with MT cells of the same glomerulus through serial synapses (blue line with dot) and reciprocal synapses (green). MT cells have AMPA and NMDA auto-receptors (shown in red) on their distal primary and secondary dendrites providing self-excitation. Granule cells connect with MT cells through reciprocal synapses. MT and granule cells interact across glomerular modules throughout the OB granule cell layer. MT cells have afferent projections to excitatory pyramidal (PYR) and inhibitory regular spiking neurons (RSNP) which are learned based on MT response patterns. MT cells connect diffusely to the PC and PYR neurons receive input from distinct glomeruli. The PC has a modular attractor memory structure with pre-wirde (non-plastic) connections from RSNP cells to PYR neurons in their respective minicolumn (MC), between PYR within one MC, from PYR to basket cells, between basket cells and feed-back inhibition from basket cells to PYR. The learned connectivity in PC includes connections from PYR to RSNP and PYR cells in other MC and vice versa providing long-range connectivity. Connections from PYR to readout neurons are learned as well. ONL, olfactory nerve layer; Glom, glomerular layer; EPL, external plexiform layer; MBL, mitral cell body layer; GL, granule cell layer. Colors represent odorants, ORN family, cell type or odor identity, respectively.

We will first describe the pathways in the OB model and explain the connectivity from OE to OB afterwards. Our model of the OB is intended to include the most prominent processing pathways and several inter- and intraglomerular interactions. The leading idea behind the synaptic organization in our OB model is to implement the hypothesized concentration interval code by MT cells within one glomerular module. As a basis for this we assume a columnar organization spanning different layers of the OB as reported by Willhite et al. (2006). For this purpose, we implement a soft winner-take-all (WTA) circuit within one glomerular module with feed-forward excitation provided by ORNs through axo-dendritic synapses, serial and reciprocal dendro-dendritic synapses between MT and PG cells and reciprocal synapses between MT and granule cells. MT cells receive direct excitation from ORNs via AMPA and NMDA receptors (Ennis et al., 1996) on their glomerular compartment resembling fast and graded monosynaptic input (Najac et al., 2011). A part of the interneurons situated in the glomerular layer (≈20% of the PG cells) also receive direct input from ORNs (Shepherd and Greer, 1998; Hayar et al., 2004; Toida, 2008). Inspired by the differences in dendritic arborization of PG cells reported by Toida (2008) we have implemented four types of PG cells that differ in their synaptic organization. Figure 1 shows a schematic of the connectivity within one glomerular module in the OB model described in the following. One type of PG cells (marked with PG_S1 in Figure 1, in Toida (2008) they are called TH-ir or type 1 neurons, as they contain the dopamine-synthesizing enzyme tyrosine hydroxylase) gets direct input from ORNs and makes a serial inhibitory (or in physiological reports often called symmetrical) synapse to MT cells. The second type of PG neurons (still being an TH-ir neuron, marked with PG_S2 in Figure 1) additionally receives dendro-dendritic excitatory input from a nearby MT cell, but inhibitis *another* MT cell as reported by Toida (2008). The third type of PG neurons (PG_R1, in Toida (2008) called type 2 neurons, CB-ir neurons as they contain calbindin-d28k, or CR-ir as they contain calretinin) lie deeper in the glomerular layer and show a different arborization pattern. These neurons form “typical” reciprocal dendro-dendritic synapses with MT cells and do not receive direct input from ORNs. The fourth type of PG neurons we implement PG_R2 has in addition to reciprocal synapses with MT neurons also inhibitory connections to other MT cells. As a rough physiological constraint we have set the number of reciprocal synapses in the glomerular layer to be about 25% (according to Shepherd and Greer, 1998).

Arevian et al. (2007) reported that lateral inhibition between MT cells with correlated activity is enhanced. We interpret this behavior as another aspect of a WTA mechanism between MT cells and use the dendritic arborization patterns of PG cells as one mechanism to implement this. Another possible mechanism underlying this lateral inhibition is the prominent dendro-dendritic inhibition between MT cells and granule cells. Granule cells make two types of reciprocal synapses, one with mitral cells from one glomerulus, the other type with MT cells from all glomeruli in the OB, hence providing interglomerular inhibition (Urban and Sakmann, 2002). As our interest lies in the function of the system, the synapse strengths have not been matched to experimental data, but have been tuned so that MT cells show the hypothesized concentration interval code within a glomerular module.

Several studies have pointed out the importance of autoreceptors in MT cells (Montague and Greer, 1999; Friedman and Strowbridge, 2000; Salin et al., 2001; Schoppa and Westbrook, 2002). We have implemented excitatory AMPA and NMDA autoreceptors on the primary dendrites and NMDA autoreceptors on the secondary dendrites of MT cells to facilitate the hypothesized WTA mechanism between MT cells through self-excitation. Despite the fact the PG cells do connect with other glomeruli, presumably via short-axon and external tufted cells we have not included this type of cells and connections here to not increase the complexity of the model even further as we wanted to explore the possibility of the concentration interval code via WTA mechanisms. Likewise, for the sake of simplicity, our OB model makes no assumptions about chemotopy in the layout of glomeruli, i.e. there is no spatial organization for glomeruli. With regard to cell populations, we have 8 mitral cells per glomerulus, 20 PG cells per MT cell, 100 ORNs per MT cell, and 200 granule cells per MT cell.

Results shown in the following are based on an OB model with 40 glomeruli, i.e. 32,000 ORNs, 320 MT cells, 6400 PG cells, and 32,000 granule cells.

#### 2.4.1. Connectivity from epithelium to OB

**Figure 2:**
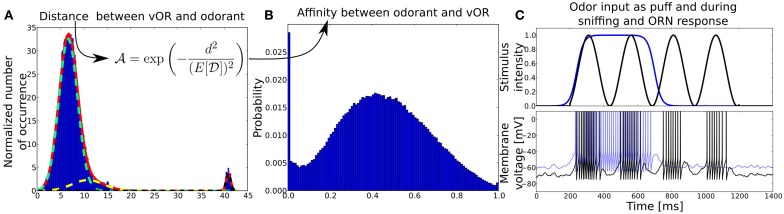
**(A)** Distribution of distances 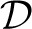 between virtual ORs and real-world odorants in a high-dimensional physico-chemical descriptor space taken from Haddad et al. (2008). Distances are obtained by clustering the multidimensional odor space a with k-means clustering algorithm, treating the resulting centroids as virtual ORs and averaging the Euclidean distances between ORs and odors over 100 trials. The red solid line shows the fit of a superposition of three normal distributions (light green, yellow, black dotted lines) to the mean distance distribution averaged over multiple clustering trials with number of ORs ranging from 20 to 66. Before the fitting, the distance distribution has been normalized by the number of ORs (centroids). The y-axis shows the normalized number of occurrence pooled over 100 trials, x-axis shows the Euclidean distance *d* in odorant space. **(B)** Affinity distribution from which affinities between odorants and receptor pairs are drawn. The y-axis shows the probability to draw an affinity given on the x-axis. The affinity distribution has been obtained by transforming the distance distribution with the given function. **(C)** Odor input (upper panel) and example membrane potentials (bottom) of an ORN to two different kind of stimuli, odor puff (in blue) and sniffing (black). The blue membrane trace in response to an odor puff is shifted by +10mV for visibility.

**Figure 3:**
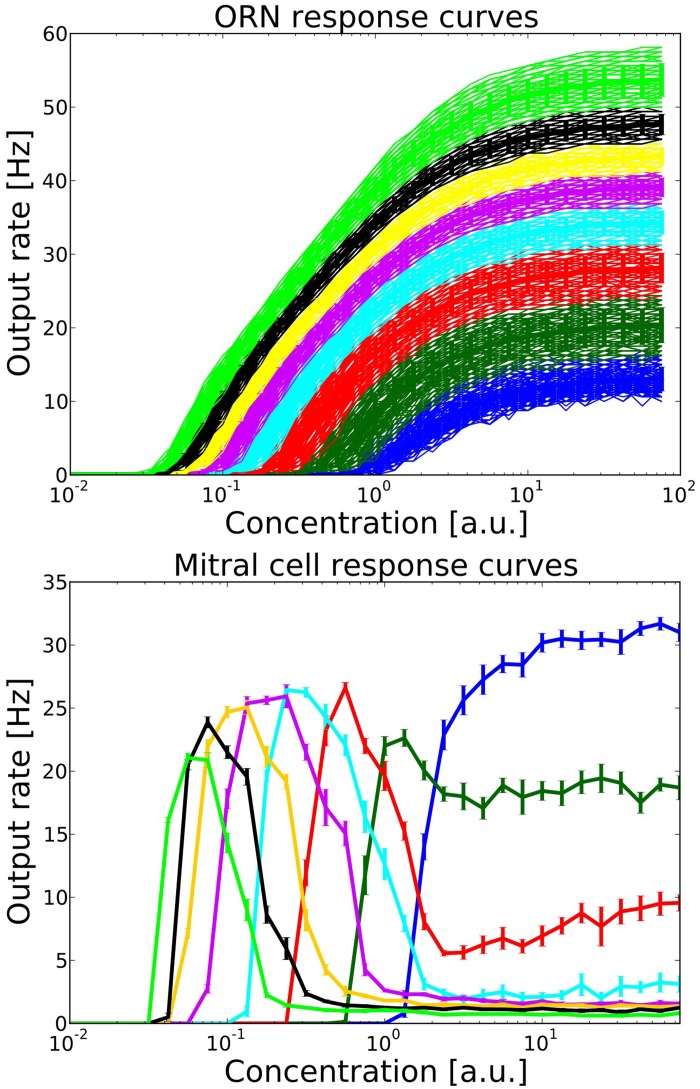
**Top:** ORN response curves from one family of ORNs expressing the same OR. Colors indicate groups within this ORN family which project to different target MT and PG cells. Each group contains 100 ORNs. Output rates were measured over one simulation run of 1600 ms, including the stimulation from one long sniff of ~400 ms **Bottom:** Mitral cell response curve averaged over 10 trials, error bars indicate the standard deviation. Colors correspond to the source group of ORNs providing excitatory input.

When connecting ORNs expressing one receptor that project to a single glomerulus, we follow the hypothesis that activity dependent axon guidance mechanisms are involved in order to create the concentration interval code in the MT population. For this purpose, we order ORNs within one family by their sensitivity and divide them into a number of different groups, each group exciting one target MT cell and inhibiting another MT cell receiving excitatory input from the next less sensitive ORN group. For example, the most sensitive ORNs respond to an odorant already at a low level of activation and activate their corresponding MT cell. The same MT cell receives inhibitory input from PG_S1 neurons that get activated by the next less sensitive group of ORNs and hence receives the equivalent of the difference of the two response curves from these two ORN groups (see Figures 2, 3 for clarification). Because of this difference in response curves exciting the MT cell, we achieve the hypothesized interval code. This effect is amplified by the inhibition each MT cell receives from PG_S2 and PG_R2 neurons (see Figure 2). The intra-glomerular inhibition provided by PG_S2, PG_R2 neurons and granule cells leads to an approximate normalization of MT activity, i.e. the output rate of a glomerulus stays approximately constant over a wide range of concentration (see Figure 3).

### 2.5. The piriform cortex

Guided by the hypothesis that the PC acts like an attractor network when learning and retrieving odor patterns, we implement the PC based on previous work as a modular attractor network (Lundqvist et al., 2006; Lansner, 2009; Lundqvist et al., 2010). Despite the fact that a modular structure based on stimulus preference comparable to orientation columns in V1, for example, has not been observed in olfactory cortices, we explore the possibility that a modular network structure as an organization principle could be involved in tasks like pattern recognition, completion and rivalry. The basic structure of our PC model consists of several computational modules [in the following called hypercolumns (HC)], each consisting of several minicolumns (MC) with 30 excitatory and 4 inhibitory cells respectively (see Figure 1 for a schematic). This modular structure has been chosen for two main reasons. First, we wanted to reflect the BCPNN algorithm as closely as possible in a spiking network in order to achieve the desired computational capabilities through attractor dynamics with soft WTA-like inhibition. Second, the modular structure including recurrent inhibition through basket cells (as described below) is required to balance excitation in the system and hence plays an important role in shaping the dynamics toward biologically plausible regimes.

Our PC model comprises three cell types that are modeled as single-compartment Hodgkin–Huxley neurons all taken from Pospischil et al. (2008). Excitatory pyramidal cells (PYR) receive input from MT cells belonging to different glomeruli (Apicella et al., 2010) and can be associated with seminlunar, superficial and deep pyramidal cells (see e.g. Bekkers and Suzuki, 2013 for a recent review of cells in the PC). Similarly to the model in Lundqvist et al. (2006), PYR cells connect to other PYR cells within the same MC with a probability of 25% and to basket cells in the same HC with a probability of 70%. Basket cells receive excitatory input from PYR cells only and connect to PYR cells in the same HC with a probability of 70% and hence provide strong feedback inhibition to PYR cells imposing a soft winner-take-all like competition among MCs belonging to the same HC. RSNP neurons receive excitation from MT cells and from PYR cells. RSNP cells project to PYR neurons belonging to the same MC with a probability of 70%. The results shown in this study are from simulations of 12 HCs with 30 MCs each, giving 10,800 PYR, 1440 RSNP, and 2160 basket cells, as we have 6 basket cells per minicolumn.

#### 2.5.1. Connectivity between OB and PC

The connectivity from the OB to PC is derived based on the mutual information between MT cells and the BCPNN algorithm, similar to previous models (Johansson and Lansner, 2006; Lansner et al., 2009). Connections are not derived on a cell-to-cell basis, but target units in the PC that are represented by MCs consisting of 30 neurons each. After the weights from MT cells to MCs have been computed they will be translated into cell-to-cell connections as described in section 2.5.3. For this purpose we simulate the responses of the epithelium and OB for 1600 ms to *N*_*p*_ = 50 different random artificial odor patterns and use the MT cell responses to calculate their mutual information.

First, the *N*_MT_ mitral cell responses to the *N*_*p*_ pattern presentations are transformed into probabilities of activation *p*_*i*_. This is done by normalizing the number of spikes *f*^*k*^_*i*_ fired by mitral cell *i* during pattern *k* by dividing through the sum of spikes fired during all *N*_*p*_ patterns:

(3) $$f_{i}^{k^{\prime }}=\frac{f_{i}^{k}}{\sum_{k}^{N_{p}}f_{i}^{k}}$$

Furthermore, we apply a half-normalization to each glomerular unit, i.e. if the summed normalized activity during one pattern in one glomerulus is higher than one, it is normalized to one:

(4) $$\xi_{i}^{k}=\{\begin{array}{ll} f_{i}^{k^{\prime }}/\sum_{i}^{q}f_{i}^{k^{\prime }} & \text{if}:\;\sum_{i}^{q}f_{i}^{k^{\prime }}>1,\\ f_{i}^{k^{\prime }} & \text{otherwise} \end{array}$$

The indices *i* and *q* stand for the MT cells belonging to one glomerulus. This half-normalization is applied because we interpret MT cells as probabilistic sensors and the normalized activities within one glomerulus as probabilities of measuring the presence of a certain feature. As MT cells code for concentration this would correspond to the probability of sensing an odorant at the corresponding concentration. This is why the normalized activities must not sum up to a value above one.

Based on the normalized activation probabilities *p*_*i*_ and probabilities for joint activation *p*_*i, j*_ are obtained by:

(5) $$p_{i}=\frac{\sum_{k}^{N_{p}}\xi_{i}^{k}}{N_{p}}$$

(6) $$p_{i,j}=\frac{\sum_{k}^{N_{p}}\xi_{i}^{k}\xi_{j}^{k}}{N_{p}}$$

Then the mutual information *I*_*i, j*_ and joint entropy *E*_*i, j*_ between mitral cells is calculated as follows:

(7) $$I_{i,j}=\{\begin{array}{ll} p_{i,j}\log(\frac{p_{i,j}}{p_{i}p_{j}}) & \text{if}:\;p_{i}\cdot p_{j}\neq 0\text{ and }p_{i,j}\neq 0\\ 0 & \text{otherwise} \end{array}$$

(8) $$E_{i,j}=\{\begin{array}{ll} -p_{i,j}\log p_{i,j} & \text{if}:\;p_{i,j}\neq 0\\ 0 & \text{otherwise} \end{array}$$

From these two quantities the mutual information distance measure is defined as:

(9) $$D_{i,j}=\{\begin{array}{ll} 1-\frac{I_{i,j}}{E_{i,j}} & \text{if}:\;E_{i,j}\neq 0\\ 1 & \text{otherwise} \end{array}$$

In order to decide which MT connects to which HC in the PC, we apply a multi-dimensional scaling algorithm (MDS) to the distances *D*_*i, j*_ into three dimensions implemented by the Python-Orange software package (Demšar et al., 2013). The mapping between MT cells and cortical HCs is achieved by doing a k-means clustering as vector quantization (VQ), resulting as HC being the centroids to a number of MT cells in the three-dimensional mutual information space. The VQ is repeated until no HC is empty, i.e. each HC gets input from at least one MT cell, ignoring MT cells that were silent during all patterns. This MT-HC mapping can be modified by allowing each MT cell to connect not only to one HC, but to the *m* nearest centroids or HCs. If not stated otherwise, we have used *m* = 4 for our simulations. A second VQ is applied to each HC to distribute the different patterns among the MCs in one HC to derive their specific response properties. This is done by building a new multidimensional MT-response space in which each mitral cell assigned to the target HC represents one dimension and each pattern represents a Euclidean vector. The normalized MT cell activation ξ^*k*^_*i*_ gives the magnitude for vector *k* in dimension *i*. The result of this second VQ maps patterns to the different MCs in a HC and gives a binary activation *N*_*p*_ × (*N*_HC_ · *N*_MC_) matrix containing information during which patterns a MC is activated by its source MT cells. This binary activation matrix is used in the next step as postsynaptic activation matrix ζ. Finally, the weights between MT cells and MCs are calculated based on the BCPNN algorithm:

(10) $$w_{i,j}=\{\begin{array}{ll} \log\frac{p_{i,j}}{p_{i}p_{j}} & \text{if}:\;p_{i}\neq 0\text{ and }p_{j}\neq 0\\ \log1/N_{p}\; & \text{if}:\;N_{p}\neq 0\text{ and }p_{i,j}=0\\ 0 & \text{otherwise} \end{array}$$

where *p*_*i*_ is the normalized pre-synaptic activation probability of MT cells, $p_{j}=\frac{\sum_{k}^{N_{p}}\zeta_{j}^{k}}{N_{p}}$ is the probability of activation of MC *j* and ζ^*k*^_*j*_ is an element from the binary activation matrix of MC *j* in pattern *k*, i.e. the information if the MC has been assigned to pattern *k* in the second VQ as described above.

#### 2.5.2. Recurrent connectivity in PC and pattern recognition readout

As before, we compute connections with the help of the BCPNN algorithm and regard MCs as elementary units and derive long-range connections between MCs belonging to different HCs based on their probability of activation in an abstract sense. The previous step gave us the projections *w*_*i, j*_ from MT cells to MCs which will now be used to calculate the responses of an abstract MC as follows. First, a MC *j* receives input *s*^*k*^_*j*_ from MT cells during pattern *k*:

(11) $$s_{j}^{k}=\sum_{i}^{N_{\text{MT}}}w_{i,j}\xi_{i}^{k}$$

This input or support is combined with the bias β_*j*_ of that MC:

(12) $$\beta_{j}=\{\begin{array}{ll} \log(p_{j}) & \text{if}:\;p_{j}>0\\ \log(1/N_{p}^{2})\; & \text{otherwise} \end{array}$$

(13) $$o_{j}^{k}=\{\begin{array}{ll} \exp\left(\beta_{j}+s_{j}^{k}\right) & \text{if}:\;s_{j}^{k}>0\\ 0 & \text{otherwise} \end{array}$$

As for MT cells that code with their normalized activity for the presence of an odorant at a certain concentration in a probabilistic fashion, we apply the same sort of half-normalization for all MCs belonging to one HC, i.e. if the sum of output activities during one pattern in one HC is larger than one, it is set to one:

(14) $$o_{j}^{k^{\prime }}=\{\begin{array}{ll} o_{j}^{k}/\sum_{j}^{q}o_{j}^{k} & \text{if}:\;\sum_{j}^{q}o_{j}^{k}>1,\\ o_{j}^{k} & \text{otherwise} \end{array}$$

The indices *j* and *q* stand here for the MCs belonging to one HC. We will come back to this point of interpreting activity as the probability of perceiving a certain feature in the Discussion.

The recurrent weights between MCs situated in different HCs is then calculated in the same way as above in Equation (10) with the output activities *o*^*k*^_*i*_ determining the probabilities of activation by replacing ξ^*k*^_*i*_ in Equations (5, 6).

MCs belonging to the same HCs are not connected. The weights within a MC (from RSNP to PYR cells and between PYR cells) are set statically and not affected by this abstract learning algorithm. The same holds for the connectivity involving basket cells.

In order to be able to classify the distributed cortical representations after learning we train an additional layer of readout cells. For training the connectivity from PC to the readout layer we use the exact same formalism, but with only one single readout cell being active during a pattern. Hence, for readout cells we set *o*^*k*^_*j*_ = 1 if *j* = *k* and 0 otherwise as a supervisor signal. This assumes that during learning the system is exposed to odorants in a pure form, in a sequential order (as separate patterns, i.e. responses are gained through separate simulations) and with the knowledge about the distinctness of odor patterns. This is also the condition for a correct recognition, when these abstract connection and bias values are transformed into the spiking network and “test patterns” are presented to the system, i.e. in the spiking context we regard a pattern as correctly classified if the corresponding readout cell has the maximal output firing rate. A readout neuron is not connected with other neurons and serves as a simple indicator if one pattern is perceived as present or not.

#### 2.5.3. Translating abstract learning results to biophysical model

As described in the above section we obtain abstract connection matrices for feed-forward connections MT cells and PC, between MCs in PCs and from the PC to a readout layer which tries to identify input patterns with the presented patterns during the training. To transform the abstract connectivity obtained from Equation (10) we do a linear mapping from the abstract weights into biophysical weights, i.e. conductance values. If the resulting biophysical weight is below a threshold of 5 pS, the connection is discarded because it has no significant influence and to decrease computational costs. For OB to PC and the recurrent PC connections, negative values get linearly mapped to positive weights that target the inhibitory RSNP cells which in turn provide inhibition to the target MC. Positive values are linearly mapped to weights that target PYR cells. Based on the source and target cell type we use different linear transformation factors, e.g. we transform negative weights so that the most negative value corresponds to a conductance of 3 nS for MT to RSNP connections and 1.5 nS when the connection originates from a PYR neuron. When an MT cell excites a MC it targets 50% of all PYR in that MC, i.e. 15 cells. When an MT cell inhibits a MC it excites 75% of all RSNP in that MC, i.e. three RSNP neurons, which in turn inhibit the PYR cells in the that MC. For recurrent PC connections, positive weights are transformed into 45 excitatory long-range connections between the two respective MCs, which corresponds to 5% of all possible connections between the two MC. Negative weights are realized so that 10 out of 30 PYR cells from the source MC target 3 out of 4 RSNP cells in the target MC. Source and target cell pairs for recurrent PC connections are chosen randomly and multiple connections between the same source and target pair are replaced with a valid source-target pair. Connecting the readout layer takes into account all PYR cells in the source MC. After the linear transformation of the abstract weights into the cell type specific conductances, all conductances on the single-cell level are randomly changed by 10% in order to account for natural variability of neurons and synapses.

The full data on resulting number of synapses and neurons in the system is shown in Table 2.

**Table 2:** **Neuron and connection numbers**.

**Neuron (connection) name**	**Type**	**Number**	**Relative connection density (%)**
ORN	Exc	32,000	–
ORN → MIT	Exc	32,000	0.3
ORN → PG	Exc	308,000	0.15
MT	Exc	320	–
MT → PG	Exc	~7360	0.36
MT → GRAN	Exc	~267,100	2.6
MT → PYR	Exc	~ 1.742 · 10^6^	5
MT → RSNP	Exc	~ 80,150	17.4
PG	Inh	6400	–
PG → MT	Inh	21,760	1
Granule cell	Inh	32,000	–
Granule cell → MT	Inh	267,100	2.6
PYR	Exc	10,800	–
PYR → PYR	Exc	~ 75,500	0.06
PYR → RSNP	Exc	1.23 · 10^6^	7.9
RSNP	Inh	1440	–
RSNP → PYR	Inh	30,240	0.2
Basket cell	Inh	2160	–
Basket cell → PYR	Inh	630	5.8
Readout neuron	Unspec	50	–
PYR → Readout	Exc	0.54 · 10^6^	100

## 3. Results

We will first show the response curves of ORNs and MT cells realizing the hypothesized fuzzy concentration interval code before we focus on the five functional tasks the system has been tested with (recognition, concentration invariance, noise robustness, pattern completion, pattern rivalry).

Figure 3 shows the output rates of one family of ORNs to an odorant to which the OR has maximal affinity for different concentrations. Output rates are measured over one full simulation of 1600 ms in response to an odor puff (see Figure 2). The response curves are color coded depending on the target MT cell to which the ORN subgroup will project according to our hypothesized axon-sorting as described in sections 1.3.1. and 2.4.1. The MT response curves are averaged over ten trials with different random seeds modifying background noise and initial membrane potentials, error bars indicate the standard deviation. Through the projection patterns described in section 2.4.1 we achieve that individual MT cells code for only a certain concentration range.

### 3.1. Task 1: basic pattern recognition

**Figure 4:**
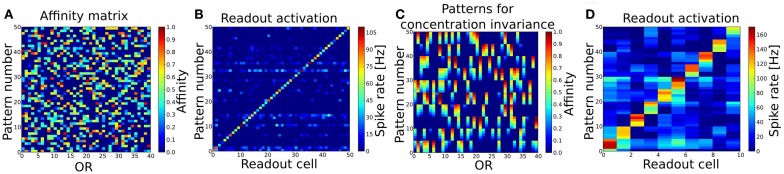
**(A)** Patterns to train and test the basic pattern recognition capabilities of the system (Task 1). 40 ORs are activated in 50 different patterns. Per pattern 30–50% of all ORs are activated. **(B)** Readout activity for pattern recognition test. As input served the 50 patterns shown in **(A)**. **(C)** Patterns to train and test concentration invariance. Shown are the first 10 patterns from **(A)** with varying concentration (affinity). **(D)** Readout activity response to the patterns shown in **(B)** after training the system with these. Independent of the concentration, all patterns get recognized correctly after the training.

The fuzzy interval code realized by MT cells is the basis for our approach of interpreting the OB as a probabilistic sensor array which provides information about certain odor features to the PC. We have tested this coding scheme and the self-organized connection algorithm first in a simple pattern recognition task (in the following referred to as Task 1). The system expresses 40 ORs and has been trained by stimulating the ORNs and OB with 50 different patterns in sequence, i.e. separate simulations using odor puffs as input. Figure 4A shows the used set of random odor patterns, which correspond to artificial odor patterns at a medium concentration.

The OB response to these 50 pattern presentations was used to derive the connectivity to and within PC and to the readout layer as described above. As a basic proof of functionality, we then presented the exact same patterns to the system again and looked at the output rates of the readout cells for each pattern (see Figure 4B). The criterion for a correctly recognized pattern is that the readout cell responsible for the given pattern as defined by the supervisor signal (see section 2.5.2) must have the highest output rate measured over the whole simulation time of 1600 ms. According to this criterion all 50 patterns have been recognized correctly.

The activity of PYR cells averaged over all 50 patterns is very sparse and distributed. During each pattern 223 ± 34 neurons (~2.0 ± 0.3%) of all neurons were active (being active measured as firing more than one spike per pattern). Still, firing rates of individual neurons could get as high as 150 Hz and mean firing rates averaged over all patterns and all cells that fired at least one spike are around 10 Hz. On average each neuron was active in only 1.0 ± 1.4 patterns (~2.0 ± 2.7%). In total 68.5% of the PYR cells were active in at least one pattern, 19.5% in more than two and 2.7% showed spiking activity in more than three patterns.

### 3.2. Task 2: concentration invariance

In order to test the system's capability of recognizing odors that appear at a different concentration, meaning that the effective activation for those OR that respond to the given odor is different, we selected the first 10 odor patterns from Task 1 and changed the affinity between an activated ORs and the odorant in five steps from −0.2 to +0.2 compared to the affinity in the training pattern (see Figure 4C). Changing the affinity is equivalent to changing the concentration as they are in our model dependent from each other *c* = OAV/(1 − OAV). This results in a set of 10 different odors with 5 different concentrations each and should be regarded as 50 test patterns. First, we tested the system as trained in Task 1 with this set of patterns and looked at the response of the readout cells. As the system was trained to distinguish 50 different odors at one single (medium) concentration pattern only (as shown in Figure 4B), the system did not recognize all patterns correctly, but three odors when presented at the lowest concentration were misclassified (data not shown). This could be interpreted as if the system would perceive these three odors at low concentration as being qualitatively different compared to higher concentrations.

Since odorants in real systems do occur at different concentrations and the perceived “effective” concentration varies during the sniffing or inhalation process, we trained the system with patterns representing odorants at different concentration. To achieve concentration invariance recognition we trained the system with the patterns representing 10 odors at 5 different concentration (Figure 4C) instead of single concentration odors only. The system then recognized the 10 different odorants correctly for all concentrations as shown in Figure 4D.

### 3.3. Task 3: noise robustness

**Figure 5:**
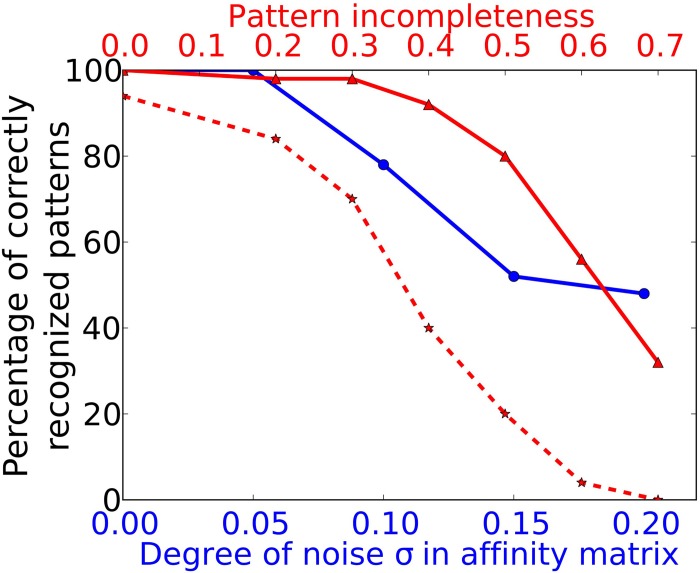
**Performance in Task 3 (noise robustness) and Task 4 (pattern completion)**. The system as trained to 50 complete and noise free patterns (as in Task 1) is exposed to odor patterns with increasing number of deactivated ORs and to patterns with increasing degree of noise. The blue curve marked with circles corresponds to the lower x-axis and shows performance in Task 3. The red curve with solid lines and triangle markers corresponds to the upper x-axis and shows performance in Task 4. The dotted red curve with star markers shows the Task 4 performance of a network without recurrent long-range connectivity in PC as trained in Task 1.

To simulate a more realistic pattern recognition task, we presented noisy versions of the 50 “pure” patterns to which the system was trained in Task 1. As described by Equation (2) we modified each element in the affinity matrix by an increasing degree of noise σ and tested the system trained from Task 1 to recognize these noisy patterns. The blue curve in Figure 5 shows the performance of the system for four different noise levels. For a degree of noise of abs(σ) ≤ 0.05 the system recognizes all patterns correctly, hence showing some noise robustness, but performance drops rapidly for larger σ. As we have chosen an extremely simple model for odorant-OR interaction without regarding possibly competitive interactions, it is not possible to relate these values to real systems in a meaningful way.

### 3.4. Task 4: pattern completion with modified temporal input

**Figure 6:**
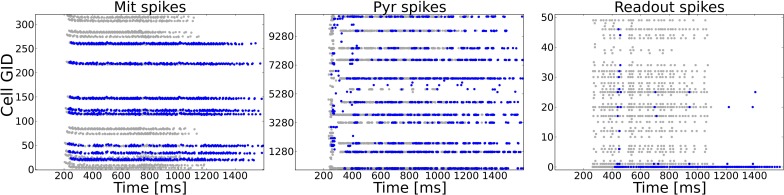
**Example activity shown as raster plots during pattern 0 in Task 4 (pattern completion)**. The system as trained to 50 complete patterns (as in Task 1) is exposed to an incomplete version of a training pattern in which 50% (randomly chosen) of the previously active ORs are silenced. The y-axis shows the cell number of the respective neuron type. Gray dots mark the activity during the training, blue dots show the activity during the test with the incomplete pattern. For the test pattern temporal dynamics of stimulation are more variable due to sniffing input as compared to the puff like input used during training. **Left:** MT spike patterns clearly show the incomplete test pattern, but only faint difference in dynamics. **Middle:** Pyramidal cells in PC show a very similar activity on the population level because of the recurrent cortical connectivity. The temporal dynamics are different as compared to the complete pattern, partly due to incompleteness but also due to the sniffing input. **Right:** The correct readout cell begins to spike approximately 150 ms later during the test pattern compared to the training pattern activity, but clearly shows higher activity than other readout cells. Hence, the incomplete pattern is correctly classified.

A typical task to be mastered by a sensory system is to deal with incomplete patterns. We model incomplete patterns by taking the system from Task 1 and choosing a random number of ORs that get activated in the complete pattern (Task 1) to be inactive in the incomplete pattern. As an additional test for the dependency of the system to rely on precise spike timings we changed the input dynamics from the odor puff (with which the system has been trained) to the more variable sniffing input (see Figure 2C). The difference in stimulation dynamics is clearly visible on the ORN level, but is less pronounced on higher levels as shown in Figure 6. This might be due to the strong influence of NMDA currents involved in feed-forward excitation, but also the self-excitation via excitatory autoreceptors on MT cell dendrites might attenuate the temporal structure imposed by the ORN layer. Figure 6 shows the activity of MT, PYR and readout cells as raster plots to one example pattern in the training and test setup with half of the ORs being silenced. The complete training pattern is plotted with gray dots, whereas the response to the incomplete test pattern is marked with blue dots. Despite the difference in temporal input structure and the fact that activity in the OB and epithelium (not shown) is significantly less, the system is able to complete the pattern in the PC. This can be seen from the fact that cells being active during training overlap with the cells active during the test to a much higher extent than it is for MT cells, where a high number of gray dots indicate the incompleteness of the test pattern. Due to the recovered activity in the PC, the PYR cells drive the correct readout cell (the lowest readout cell, as it was pattern 0).

We have studied this pattern completion capability in a more systematic way by testing all patterns trained during Task 1 with different levels of completeness. Pattern completeness is defined by the fraction of ORs being active in the test pattern compared to the number of activated ORs in training patterns. Pattern completeness was varied from 80% to 30% and the number of correctly recognized patterns was counted as shown by the red trace in Figure 5. For all test patterns in Task 4 and 5 we used the sniffing input model in contrast to the training runs which uses an odor puff as input (see Figure 2).

The systems seems robust toward incomplete patterns for missing up to 40% of the odor components as the recognition performance stays above 90%. As shown by the example raster plots in Figure 5, the activity pattern in the PC seems very similar on the population level in the sense that the same MCs are active in comparison to the activity evoked during training, despite the missing odor information and the different temporal structure. This pattern completion capability is presumably due to the recurrent excitatory cortical connections which help to restore activity in MCs that receive less input from the OB during incomplete patterns. In order to prove this assumption we have tested a network with the exact same patterns but removed the long range connections between PYR and RSNP that have been trained during Task 1. The result is shown by the dotted red line in Figure 5. It shows a drastically impaired performance compared to an “intact” network with trained long-range connectivity between HCs.

### 3.5. Task 5: pattern rivalry with modified temporal input

Perceptual bistability or rivalry occurs when stimulus patterns overlap so that two distinct perceptions are possible. This phenomenon can be explained by attractor dynamics in the networks involved in sensory integration and perception. We have investigated the system's response behavior to odor patterns that are constructed from varying subparts of distinct patterns from Task 1.

In order to study the system's responses to mixtures, we constructed new odor patterns by choosing a pair of two of the 50 distinct odors patterns, with which the system has been trained in Task 1, and generating a new set of patterns by varying the number of components taken over from the respective two patterns. For example, a mixture of 0.4/0.6 between two arbitrary patterns B and R is built by choosing randomly 40% of the ORs active in pattern B and 60% or ORs activated in pattern R and combine them into a mixture pattern. This has been done for 50 different pattern pairs with a varying fraction of each pattern from 0.8/0.2 to 0.2/0.8 in steps of 0.1, taking over the previously chosen ORs into the next mixture pattern resulting in a sequence of mixture patterns which morphs from one to the other. This gave us seven different mixture patterns for each of the randomly chosen pure training pattern pair. We chose to pick 50 different odor pairs to generate in total 350 mixture patterns. This large set of mixture patterns has been presented to the system that has been trained with pure patterns as in Task 1 in sequential order.

**Figure 7:**
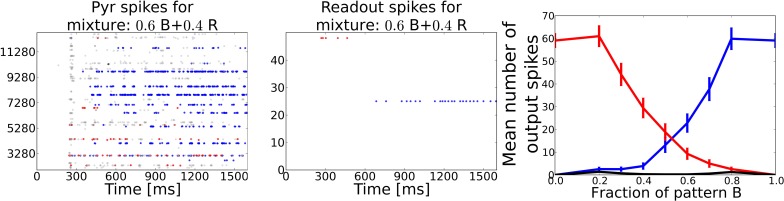
**Task 5: Pattern rivalry with sniffing input**. **Left:** Raster plot showing PYR responses to a 0.6/0.4 mixture of two distinct patterns. The fraction of both patterns stays constant during the whole stimulation. Blue dots show spikes from cells being active during the “blue” odor in Task 1. Red dots show spikes from cells being active during the “red” odor in Task 1. Gray dots show spikes from cells that are active during the test pattern, but have not been active in either of the two mixture components. During the first 200 ms the red pattern evokes activity in both PYR and readout cells, but is then suppressed by the blue pattern becoming active after ~500 ms of odor stimulation. A substantial part of PYR activity is related to none of the two patterns, exemplary for the often occurring misclassifications during the recognition of odor mixtures. **Middle:** Raster plot corresponding to the pattern from left panel showing spikes emitted by readout cells that were active during the two respective training patterns. The stronger pattern is being recognized starting from ~700 ms, i.e. approximately after 500 ms or two sniff cycles (simulated sniffing frequency is around 4 Hz. **Right:** Average curves showing the mean number of spikes emitted by the readout cells trained to recognize one of the two test patterns. Black curve shows the mean response from readout cells that code for none of the two test patterns. The blue and the red curve indicate a smooth transition from one pattern to the other depending on the relative strength in the mixture.

We counted the number output spikes by the readout neurons corresponding to the two unmixed training patterns and averaged these over the 350 mixture patterns (see Figure 7). The average curve shows a smooth transition from one pattern to the other and rivalry behavior in between, meaning that the system recognizes both patterns at the same time (regarded over one pattern presentation). During the morphing process from one pattern into the other it often occurred that the readout layer recognized none of the two partial test patterns but interpreted the superposition as a different pattern.

When looking at the dynamics during a single example mixture as shown in the left two panels of Figure 7, the PC and the readout activity indicate two distinct odor percepts (as indicated by the color of the dots) at different times during the stimulation. Hence, the systems perception switches dynamically from one odor to the other which is characteristic for perceptual rivalry.

## 4. Discussion

In this study we have presented a generic architecture for self-organized pattern recognition and memory systems and implemented a spiking model thereof inspired by the first three stages of the mammalian olfactory system. We have proven the functionality of the system in different pattern recognition tasks involving concentration invariant recognition and pattern completion, and studied its robustness against noise and rivalry phenomena occurring with mixtures of odor patterns. Our approach is generic, because it can be used for other modalities as well (Lansner et al., 2009), as the format of the sensory array on which the learning algorithm operates is modality specific, but the cortical structure responsible for the integration and consolidation of sensory information is regarded to be modality independent.

### 4.1. Original hypotheses

One of our key hypotheses is that activity dependent axon sorting mechanisms contribute to the formation of a concentration interval code in the MT layer of the OB. The motivation behind the hypothesized interval coding is to use the OB as a probabilistic sensor array that serves as input for the BCPNN algorithm which allows for self-organization of the connectivity from OB to PC and within the PC based on the probabilistic interpretation of MT responses. Here, we explored the possibility of such a coding scheme in the context of odor concentration and showed that it is implementable in a spiking context.

Furthermore, we assume that the PC acts similar to other cortices as an attractor network and hence applied a modular network structure to simulate functions like pattern completion and rivalry. We are well aware of the fact that no columnar organization has been reported in olfactory cortices and we suggest that the computational structure is not necessarily visible from the spatial layout of cells as in other sensory systems, e.g. in V1 (Li et al., 2012), but rather implemented through the connectivity patterns, e.g. MCs could correspond to small, spatially dispersed populations with enhanced recurrent connections that connect to a common pool of inhibitory interneurons (corresponding to basket cells in our model). A softening of the rule that basket cell inhibition targets only PYR belonging to the same HC was investigated in studies by Lundqvist et al. (2010, 2013). There the modular basket cell inhibition was replaced by a distance dependent inhibition and it was shown that the network dynamics change, but that the attractor behavior, and with that the computational capabilities of the system, are preserved without this modular inhibition. The computational capabilities of the presented model are rather based on the specific long-range excitation between MCs and the specific inhibition mediated through RSNP cells (Fransén and Lansner, 1998), whereas basket cell inhibition is required to regulate the network activity and balance the excitation. This is because RSNP cells in our model can not counterbalance the recurrent excitation within an attractor.

The strict columnar organization as used in our model was chosen to reflect the BCPNN algorithm more closely, but is likely softened in real systems. Hence, in this respect our network model should not be seen as a precise model of the biological counterpart but rather as a way to implement networks performing holistic computations and behaviorally relevant functions. One advantage of the modular structure and the assumed patchy connectivity is a shorter wiring length with the same pattern storage capacity when compared with a non-modular “pepper-and-salt”-like organization (Meli and Lansner, 2013).

### 4.2. Summary of findings and explanation

We have shown that the self-organization algorithm previously used only in abstract models (Lansner et al., 2009; Persaud et al., 2013) can be translated into a spiking network context, and that pattern recognition can work on the time scale of a single sniff, comparable to results from behavioral studies (Uchida and Mainen, 2003). First of all, we have shown that a concentration interval code can be implemented with the help of known pathways in the OB with biophysically detailed neuron and synapse models. Furthermore, we have successfully translated an abstract self-organization framework to a spiking network and shown its functionality in a simple pattern recognition task (Task 1). The key components to achieve this functionality is the projection from OB to PC and the connectivity within PC obtained from the BCPNN algorithm. The system has proven to be robust against changes in temporal dynamics and high levels of incompleteness at the same time. In a pattern completion task we have shown that the recurrent excitatory connectivity in the PC promotes the restoration of incomplete pattern activity and facilitates pattern recognition of incomplete patterns.

In addition, we have shown that concentration invariant recognition emerges after training the system with patterns at multiple concentrations. This brings us to the conclusion that concentration invariance could be learned through experience by exposure to odorants that effectively always vary during the sniffing or inhalation. A system without having been trained to perceive odors of different concentrations as belonging to the same odorant can lead to qualitative different percepts as we observed in our simulations, and has been reported for some odorants (Gross-Isseroff and Lancet, 1988; Johnson and Leon, 2000; Wright et al., 2005).

Surprisingly, little differences in bulbar and cortical activity were observed when different stimulation protocols were applied. One possible explanation for this could be that NMDA currents dominate the behavior more than fast excitatory currents do, as NMDA currents are found almost ubiquitously in the system, e.g. as source for self-excitation in MT cell dendrites. Thus, we conclude that the input dynamics including precisely timed spike patterns or sequences thereof do not play a crucial role for the pattern recognition capabilities of our model system. It remains subject to debate whether this finding can be seen as an argument against spike timing dependent codes, as different stages might use different ways of coding as suggested by Haddad et al. (2013). Similarly, other concentration coding schemes as the one used in our model could work equally well.

### 4.3. Results in context to other existing studies

In general our results are in qualitative agreement with recent experimental findings regarding odor representations in the PC and projections from OB to PC. The connectivity obtained by our self-organization method leads to PYR neurons that integrate information from distinct glomeruli as seen in Apicella et al. (2010). Furthermore, we observe sparse and distributed activity in PC in response to odor stimuli with activation levels in a comparable range to findings by Stettler and Axel (2009). In accordance with (Poo and Isaacson, 2011) we observed rather unspecific inhibition in PC, as connectivity involving basket cells is not dependent on the source or target cell's response properties. In addition, weights from OB to PC observed after training are often inhibitory and hence provide inhibition for a large number of odorants.

### 4.4. Limitations

Despite the complexity of the presented model, there are a large number of limitations and aspects which have not been covered at all by our model. Regarding the general (structure), our model does not include any notion of the anterior olfactory nucleus (Brunjes et al., 2005), and other input sources into PC from other areas than the OB were not regarded in our model (see e.g. Luna and Morozov, 2012). Differences between the anterior and posterior PC have not been included in the model as well as learning in other structures (Morrison et al., 2013). Our model does also not include neurogenesis seen in the OB of rodents (Nissant et al., 2009; Sahay et al., 2011), but whether neurogenesis is crucial in the human olfactory system is still up for debate (Bergmann et al., 2012). Acetylcholine was not included in this model, but the role of cholinergic modulation might impact memory performance as shown in de Almeida et al. (2013). More specifically, our implementation of the concentration code is not easily extendable to larger neuron numbers, as this would require substantial retuning of various parameters to achieve the desired response curves.

One very important limitation of the model, as was presented here, is the lack of projections from the PC to the OB. The back-projections do play an important role in odor recognition, especially in tasks where attention or the expectation of an odor changes the signals represented in the system. This task-relevant information could be included in an extension of our model using external input into the PC and the inverted OB to PC weight matrix, which would make PYR neurons target granule cells, preferably connecting the respective glomerular module, so that task-relevant information acts like a template on the bulbar layer to filter or enforce certain patterns.

### 4.5. Outlook

In general, two broad directions could be taken starting from the presented model. One is making the model more realistic and trying to verify or falsify it, e.g. by using more realistic odor patterns, incorporating more experimental data specifying the circuits involved, adding cell types and structures that have been omitted in this model. The opposite direction is to simplify certain components even further (e.g. reducing the complexity of ORNs, and bulbar cell models) and test the model in different and more complex tasks, e.g. odor segmentation. The question on which scale the inhibition in cortical circuits acts in a computational meaningful manner, in our model represented by the size of a hypercolumnar module, and how the extent of this recurrent inhibition is sensitive to feed-forward excitation and the spread thereof is unknown and needs to be investigated in the future. As this study is only a first step in transforming abstract learning paradigms into the context of functional spiking network models and thereby trying to bridge the gap between system-level functions and biophysical detail, this model offers the possibility for versatile extensions and improvements, to be examined in future studies.

### Conflict of interest statement

The authors declare that the research was conducted in the absence of any commercial or financial relationships that could be construed as a potential conflict of interest.

## Abbreviations

OR: olfactory receptor
ORN: olfactory receptor neuron
OB: olfactory bulb
MT: mitral or tufted cell
PG: periglomerular cell
PC: piriform cortex
PYR: pyramidal neurons
AMPA: α-amino-3-hydroxy-5-methyl-4-isoxazolepropionic acid
NMDA: N-methyl-D-aspartate
GABA: γ-aminobutyric acid
WTA: winner-take-all operation
MC: minicolumn
HC: hypercolumn
VQ: vector-quantization
MDS: multi-dimensional scaling.
